# Utilization of Spent Coffee Waste Biomass as a Promising Feedstock in Bioplastics Production Using *Cupriavidus necator*

**DOI:** 10.3390/polym18161945

**Published:** 2026-08-08

**Authors:** Ganesh Saratale, Rijuta Ganesh Saratale, Ram Naresh Bharagava, Anil Kumar Patel, Dong Su Kim, Ramesh Kumar, Han Seung Shin

**Affiliations:** 1 Department of Food Science and Biotechnology, Dongguk University-Seoul, Ilsandong-gu, Goyang-si 10326, Republic of Korea; 2 Department of Earth Resources & Environmental Engineering, Hanyang University, Seoul 04763, Republic of Korearameshibt@gmail.com (R.K.); 3 Department of Environmental Microbiology, School for Environmental Sciences, Babasaheb Bhimrao Ambedkar University, Lucknow 226 025, Uttar Pradesh, India; 4 Institute of Aquatic Science and Technology, College of Hydrosphere, National Kaohsiung University of Science and Technology, Kaohsiung City 81157, Taiwan; 5 Department of Environmental Science and Engineering, Ewha Womans University, Seoul 03760, Republic of Korea

**Keywords:** spent coffee ground, alkaline pretreatment, enzymatic hydrolysis, *Cupriavidus necator*, polyhydroxybutyrate (PHB), corn steep liquor

## Abstract

The increasing demand for sustainable bioplastics is constrained by the high production costs associated with refined carbon sources, highlighting the need for low-cost, renewable feedstocks. This study evaluated the potential of spent coffee grounds (SCG), an abundant and sustainable feedstock for generating polyhydroxyalkanoates (PHA) using *Cupriavidus necator*. First, SCG was subjected to solvent extraction to remove coffee oil, followed by extraction of phenolic compounds, and the remaining biomass was referred to as SCGO. The originality of this work lies in the systematic comparison of acid, alkaline, and peracetic acid pretreatments and their subsequent evaluation for microbial PHA production. SCGO was subjected to various chemical pretreatments, including acid (H_2_SO_4_), alkaline (NaOH), and peracetic acid (PAA) pretreatment. The effects of the different pretreatments on SCGO delignification, hydrolysis yield, and enzymatic saccharification to release monomeric sugars were evaluated. Among the tested methods, alkaline pretreatment provided the highest delignification efficiency, enzymatic saccharification, and fermentable sugar recovery, resulting in superior bacterial growth and PHA production. Under optimized conditions, the alkaline-pretreated SCGO hydrolysate supplemented with corn steep liquor produced a maximum biomass concentration of 6.5 ± 0.26 g/L, 60.0 ± 1.45% PHA accumulation, and a PHA titer of 3.89 ± 0.14 g/L. Structural and thermal characterization confirmed that the produced polymer possessed properties comparable to those of conventional poly(3-hydroxybutyrate) (PHB). Overall, this study demonstrates that integrated valorization of spent coffee grounds can effectively generate fermentable substrates for microbial PHA production, providing a sustainable approach for converting agro-industrial residues into high-value bioplastics while supporting circular bioeconomy strategies.

## 1. Introduction

The increase in population and standards of living has sparked a drastic surge in global energy consumption, raising concerns about the use of fossil fuels. Plastics have been important in improving the quality of life and social well-being for all of humanity since the 1950s [1]. Their versatile nature, low price, and ease of processing make them widely used across a range of industries [2]. Nevertheless, reliance on non-renewable sources, inadequate sustainability, and limited degradability have rendered plastics a significant environmental concern and created a pressing need to develop sustainable waste management practices. The increasing accumulation of plastic waste has become a significant global environmental challenge, adversely affecting terrestrial and aquatic ecosystems while also posing potential risks to human health through the persistence and accumulation of plastic debris and microplastics in the environment [3]. Polyhydroxyalkanoates (PHA) are intracellularly synthesized polymers that microbes use as energy storage under nutrient-limited conditions. PHA are emerging as promising alternatives to petroleum-based plastics due to their biodegradable properties, flexibility, and sustainability [4]. These biodegradable polyesters have extensive applications in the food industry, medicine, agriculture, and cosmetics, and have economic, environmental, and social advantages [5]. PHA can be completely biodegraded into nontoxic substances, making them environmentally friendly [6,7]. The global production capacity of bioplastics reached 2.31 million tonnes in 2025. PHAs have been produced on industrial scales of 1000–10,000 tonnes per year in major companies around the world, such as Biomer (Germany), Bio-On (Italy), CJ Bio (South Korea), Danimer Scientific (USA), TianAn Biologic Materials Co. (China), and RWDC Industries Ltd. (Singapore) [8]. Nevertheless, the production cost of PHAs is much higher than that of petrochemical plastics, which further impedes their large-scale commercialization [9,10,11].

The general sources of carbon used for PHA production are refined carbohydrates, fatty acids and derivatives, and alkanes. The use of these relatively high-cost substrates greatly increases overall production cost and reduces the economic viability of large-scale PHA production [12]. Substrate selection is equally a critical economic consideration because media expense accounts for close to half of the overall PHA production cost. Thus, studies aim to develop more cost-effective and environmentally friendly processes by using various waste products as low-cost carbon sources to produce PHA [13]. In addition, the selection of a suitable bacterial strain is also critical in PHA production. The suitability of an organism for large-scale polymer production depends on several factors, such as the cell’s capacity to utilize a waste carbon source, growth rate, polymer production rate, integrated metabolic pathways, and the maximum amount of polymer that can be accumulated. *Cupriavidus necator* is one such strain attributed to the production of mcl-PHA because it can efficiently utilize various renewable waste materials and efficiently utilize five- and six-carbon sugars. The biosynthetic pathway mainly involves β-oxidation via several enzymes, such as PHB synthase and acetoacetyl-CoA synthetase, which facilitate the conversion of acetyl-CoA to PHB [14].

Coffee has been consumed for more than 1000 years, with current annual consumption exceeding 400 billion cups [15,16]. It is also the world’s second-most traded commodity after crude oil and its derivatives. The International Coffee Organization reports that the world output of coffee has grown by 59.3% over the last 20 years and is currently 169.34 million 60 kg bags per year [16,17,18,19]. The main significant environmental issue associated with coffee consumption is the production of huge quantities of spent coffee grounds (SCG). Around 0.65 kg of SCG per kilogram of instant coffee is produced after brewing. It is estimated that approximately 60 million tonnes of SCG are produced annually worldwide [20,21]. Most SCG is disposed of as waste, which is typically sent to landfills or incinerated [22]. Landfill disposal contributes to methane production and soil acidification because SCG has high organic matter and lipid contains more organic matter and lipids, and incineration produces harmful gases that contribute to global warming, including nitrogen oxides and carbon monoxide [18]. These effects exert tremendous strain on waste management frameworks owing to the high quantity of contaminants produced by existing SCG disposal processes [23].

To alleviate these difficulties, extensive research has been dedicated to valorizing SCG into value-added products. The major constituents of SCG are carbohydrates (58–60%), proteins (13–18%), and lipids (12–17%), together with high-molecular-weight melanoidins, alkaloids, and phenolic compounds [24,25,26]. Research efforts have mainly focused on biofuel production (biodiesel, bioethanol, and biogas), enzyme and biopolymer production, and pharmaceutical and nutraceutical formulations. Moreover, their potential applications in agriculture and the environment have been explored to support the development of a bio-based economy in the coffee industry [18,24]. The number of scientific publications on SCG has increased significantly over the past 15 years, reflecting its broad potential for industrial applications. This trend highlights the growing importance of SCG valorization as a research topic. For the commercial implementation of SCG-based biorefineries, feedstock logistics, storage requirements, and supply-chain considerations are important aspects.

Beyond the above processes, SCG also has potential as a low-cost, renewable feedstock to produce valuable PHA and minimize environmental pollution. The biorefinery process comprises three main stages: (i) pretreatment, (ii) fermentation, and (iii) product recovery. SCG consists of a mixture of amorphous and crystalline cellulose embedded within a hemicellulose–lignin matrix. Lignin also provides resistance to chemical and biological decomposition due to its physical structure [27], which restricts biological degradation and causes low conversion efficiencies of organic matter to fermentable substrates [27,28]. Such structural complexity limits enzyme accessibility. Thus, to enhance the conversion of the SCG to PHA, it is imperative to develop efficient and effective pretreatment strategies to enhance enzymatic hydrolysis and fermentation. Traditional pretreatment methods involve chemical, thermal, mechanical, and biological treatments, which have been shown to boost yields of fermentable sugars by lowering crystallinity and polymerization levels, breaking lignin–carbohydrate complexes, and augmenting biomass porosity [29].

Among chemical techniques, the simplest and most studied is sodium hydroxide (NaOH) pretreatment, which has high delignification efficiency and a short retention time. Alkaline pretreatments (KOH or NH_4_OH) remove lignin efficiently while preserving carbohydrates, improving enzymatic digestibility with minimal inhibitor production [30,31]. NaOH is efficient in solubilizing lignin, making it porous, and resulting in swelling of the fibers [28]. In addition, peracetic acid (PAA) serves as a strong oxidizing agent that breaks the structure of lignin, splitting side chains and β-aryl ether bonds and oxidizing aromatic units of lignin. The resulting depolymerized lignin fragments, which get depolymerized, are water-soluble, thereby improving the accessibility of holocellulose for enzymatic hydrolysis [32,33]. Acid hydrolysis is a simple and efficient pretreatment that provides fast reactions, low-cost catalysts, and simple operating conditions.

Nevertheless, the concentrations of organic acids and furan derivatives affect enzymatic hydrolysis as well as microbial fermentation. Hemicellulose could be extracted efficiently using H_2_SO_4_, with a maximum sugar recovery of 70–90%, and the aqueous extract contains inhibitory compounds including furfural, hydroxymethylfurfural (HMF), and phenolic compounds. Phosphoric acid (H_3_PO_4_), frequently used for pretreatment, favors cellulose swelling with less inhibitor formation but without sufficient delignification efficiency [30,31]. Therefore, it is essential to use appropriate acid concentration to make the process more efficient and effective [34,35]. AFEX achieves high sugar conversion (80–95%) with negligible inhibitors but requires high-pressure equipment and ammonia recovery [36,37]. Hence, alkaline pretreatment was chosen in this study because of its good delignification action, fewer inhibitors, and suitability for microbial fermentation. Finally, the application of such hydrolysates to microbial fermentation for PHA production is highlighted.

**Figure 1:**
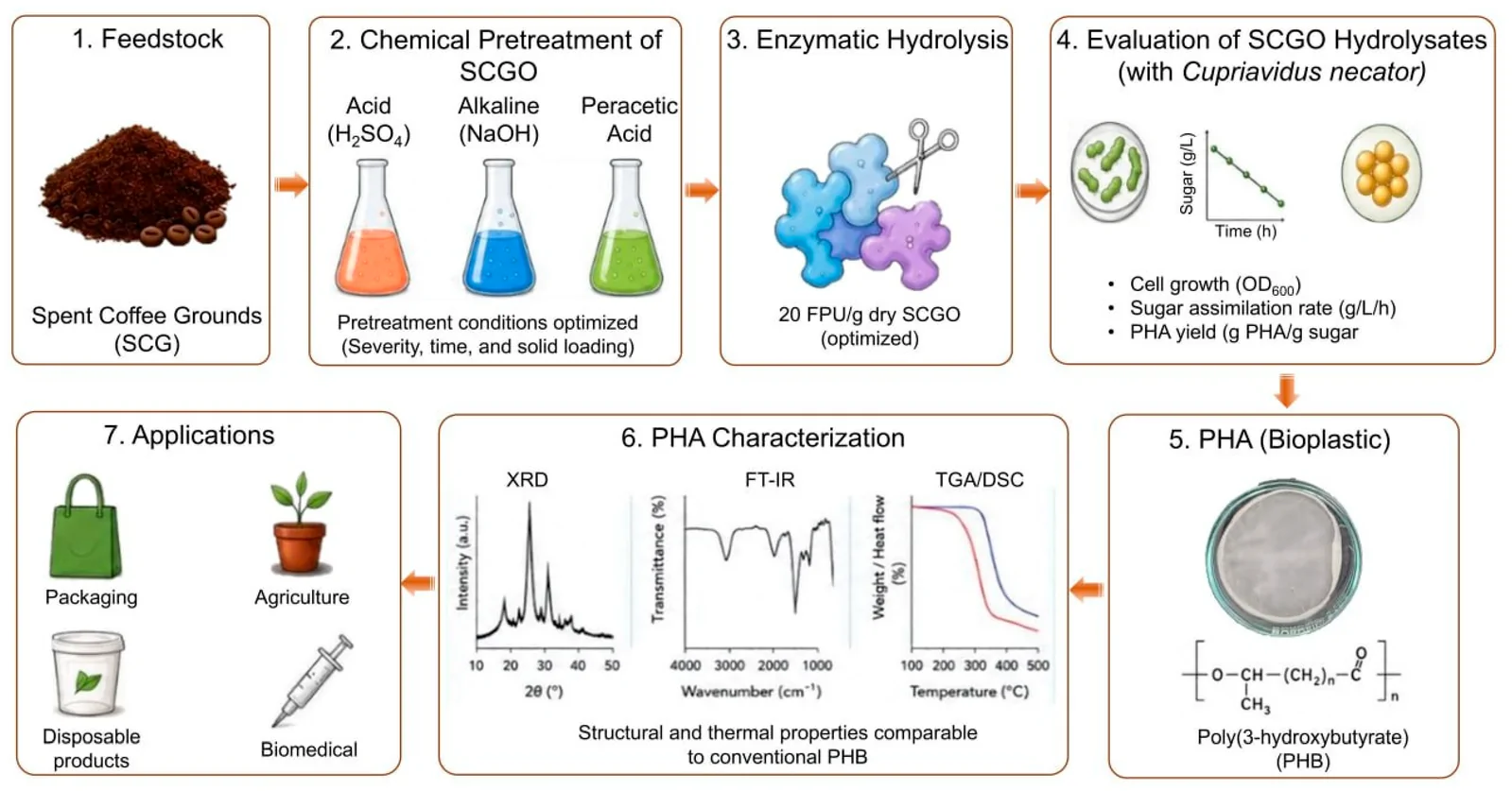
Schematic representation of the proposed research work for the development of SCG to PHA conversion process.

In previous studies, spent coffee grounds (SCG) were mainly used directly for biofuel or biochemicals production, while, in this study, defatted spent coffee grounds (SCGO) obtained after sequential extraction of coffee oil and phenolic compounds were used. The integrated biorefinery yields comprehensive valorization of SCG by converting the residual lignocellulosic biomass into polyhydroxyalkanoates (PHA). This study critically compares the various chemical pretreatments tested using defatted SCG, namely acid, alkaline, and peracetic acid. A systematic comparison of the three different pretreatment methods is carried out for their efficiency in improving enzymatic saccharification and hydrolysate fermentability. In addition, various fermentation conditions, such as substrate concentration, nutrient supplementation, and stress induction, were optimized to increase PHA production using *C. necator* and alkaline pretreated SCGO enzymatic hydrolysates. The produced PHA were further characterized for their physicochemical and thermal properties using various analytical techniques. This study provides a sustainable pathway for the valorization of SCG within the circular bioeconomy framework and reduces the environmental consequences associated with the coffee industry. Figure 1 presents the schematic illustration of the conceptualized research study.

## 2. Materials and Methods

### 2.1. Collection and Preparation of SCG Feedstock and Microbial Strain

Single sampling of fresh spent coffee grounds (SCG) was collected from a cafe at Dongguk University, Republic of Korea, and then stored at −20 °C until use. The SCG were dried in the oven at 105 °C (24 h). Oil extraction was carried out using a Soxhlet apparatus, with n-hexane as the solvent due to its high extraction capability. In short, 25 g of dried SCG was introduced into a pre-weighed cellulose thimble and subjected to 200 mL of *n*-hexane for 1.5 h at 70 °C. The solid residue was dried in a fume hood for 24 h after extraction and weighed. Solvent evaporation was used to concentrate the oil–solvent mixture by rotary evaporation at 50 °C and a rotary evaporator speed of 50 rpm for 30 min. The following equation was used to determine the SCG oil yield.

(1) $$SCGs\;oil\;extraction\;yield\;(\%)=\frac{SCGs\;weight\;change}{SCGs\;original\;weight}\times 100$$

The designated SCG biomass, referred to as (SCGO), was dried and stored in sealed plastic bags under refrigerated conditions until required. The solutions in all experiments were made using double-distilled water (DDW, Millipore, Billerica, MA, USA). Chemicals used for oil extraction and chemical pretreatment, PHA production, and extraction were of analytical grade. The bacterial strain *Cupriavidus necator* ATCC17699 was acquired from the American Type Culture Collection (ATCC, Manassas, VA, USA). The bacterial strain was further cultured in Tryptic Soy Broth without dextrose (TSB; Becton Dickinson, Franklin Lakes, NJ, USA) and stored at 4 °C. Periodic sub-culturing (monthly) was done to maintain the culture viability.

### 2.2. Chemical Pretreatments of SCGO

Before chemical pretreatment, polyphenols were extracted by subjecting 20 g of SCGO to 40 mL of 80% (*v*/*v*) methanol at 50 °C under continuous stirring (150 rpm). The biomass after extraction was filtered, dried, and then subjected to further chemical pretreatment. The dried SCGO biomass was pretreated with alkaline (2% *w*/*v* NaOH), acidic (2% *v*/*v* H_2_SO_4_), and freshly prepared peracetic acid (2%) and then incubated in an electric water bath at 100 °C for three hours. The solid-to-liquid ratio was maintained at 1:10 (*w*/*v*) in each treatment. After pretreatment, the reaction mixtures were centrifuged at 4000 rpm for 20 min (Labogene 1736R, Lillerod, Denmark) to separate the solid fraction. The pretreated SCGO biomass was then washed thoroughly using tap water until the biomass was found to be at neutral pH (pH 7.0) and, finally, using a hot air oven, the biomass was dried at 60 °C until a constant biomass weight was attained. The samples were dried and then used in the enzymatic saccharification experiments.

For better hydrolysis of SCGO, the effects of various NaOH concentrations (1–4%), SCGO substrate loadings (5–12.5%), incubation temperatures (room temperature (RT), 30 °C, 80 °C, 100 °C, and autoclaving at 121 °C for 15 min) and pretreatment times (2–6 h) were evaluated using a one-factor-at-time (OFAT) approach while keeping the other experimental conditions constant. The optimization of the parameter was assessed based on delignification, total reducing sugar production yield, and hydrolysis yield of SCGO after enzymatic hydrolysis (20 FPU/g of SCGO).

### 2.3. SCGO Enzymatic Hydrolysis Using a Produced Crude Enzyme Cocktail

Enzyme hydrolysis was carried out on chemically pretreated SCGO using a mixture referred to as a crude enzyme cocktail formed by the *Streptomyces* sp. MDS during solid-state fermentation of wheat straw under optimum growth conditions previously described [38]. Whatman filter paper was used to measure total cellulase activity [39] as per the IUPAC protocol in filter paper units (FPU), in which one unit of cellulase was equivalent to the release of 1 mmol of reducing sugars per minute. The enzymatic hydrolysis process was performed on untreated and chemically pretreated SCGO (2.0% *w*/*v*) in citrate buffer (50 mM, pH 5.0) in the presence of sodium azide (0.005% *w*/*v*) to prevent microbial growth. The reaction mixture was incubated at 50 °C for 24 h in an orbital shaker operated at 150 rpm. The aim was to assess the performance of the produced crude enzyme complex at varied substrate concentrations (5–20 g/L) by keeping constant enzyme dosage (20 FPU/g). In another series of experiments, the enzyme loading was 15–30 FPU/g at a constant SCGO concentration (10 g/L). The reducing sugar released was used to determine the hydrolysis efficiency, and the hydrolysis yield was calculated as mentioned earlier [38].

(2) $$Hydrolysis\;yield\;(\%)=\frac{Reducing\;sugar\;\left(\mathrm{mg}\right)\times 0.9\times 100}{Holocellulose\;content\;in\;the\;substrtate}$$

where 0.9 is the correction factor (conversion factor) used to account for the increase in mass when cellulose is hydrolyzed into glucose (glucose has a higher molecular weight than the anhydroglucose unit in cellulose).

### 2.4. PHA Production Studies Using Chemically Pretreated SCGO

*Cupriavidus necator* ATCC17699 was first tested in terms of production of polyhydroxyalkanoate (PHA) with enzymatic hydrolysates of acid-, alkaline-, and PAA-pretreated SCGO as carbon sources [40]. These hydrolysates were used to determine PHA accumulation and *C. necator* growth. The PHA production medium was a specific mixture of mineral salt solution that contained (g/L): NaH_2_PO_4_, 3.6; K_2_SO_4_, 3.486; Na_2_HPO_4_, 2.84; NaOH, 0.4; (NH_4_)_2_SO_4_, 0.1; MgSO_4_·7H_2_O, 0.39; MnSO_4_·H_2_O, 0.024; CaCl_2_, 0.062; CuSO_4_·5H_2_O, 0.005; FeSO_4_·7H_2_O, 0.15; yeast extract, 0.2; and ZnSO_4_·7H_2_O, 0.024, adjusted to pH 7.0. PHA production was initiated by adding enzymatic hydrolysates of SCGO after each pretreatment in 100 mL medium in Erlenmeyer flasks (250 mL) to obtain an initial sugar concentration of about 20 g/L. The inoculum was prepared by cultivating *C. necator* in Tryptic Soy Broth until it reached the exponential growth phase. The production media were inoculated with 5% (*v*/*v*) of a vigorously growing culture of *C. necator*. Optimized batch fermentations were performed at 30 °C with agitation at 200 rpm and pH 7.0 up to 48 h. These operating conditions were constant in all PHA production experiments.

The influence of the alkaline-pretreated (2% NaOH) SCGO hydrolysates at the concentration levels 10, 20, 30, and 40 g/L on the production of PHA using *C. necator* was examined. Nutrient supplementation was also tested to increase cell growth and PHA accumulation. In particular, the effects of complex nutrient sources, including yeast extract, peptone, ammonium sulfate, corn steep liquor (CSL), and ground nut cake (GNC), at 1% (*w*/*v*) concentration were examined. The effects of external stress were evaluated by adding ethanol and methanol at 1% (*v*/*v*) concentration, and the effect of osmotic stress through the addition of NaCl (5, 10, and 15 g/L) on cell growth and PHA accumulation. Experiments were conducted in triplicate to ensure reproducibility. Bacterial growth and PHA production were monitored according to the previously described procedures [40]. Further, dry cell weight (DCW) was calculated by centrifuging the culture; the obtained cells were washed with hexane and distilled water, the cell pellets were weighed, and the samples were lyophilized. The parameters for cell growth and PHA production were subsequently calculated using the formulas described earlier.

(3) $$Residual\;biomass\;\left(g/L\right)=Drycell\;weight\;\left(DCW\right)-Extracted\;quantity\;of\;PHA\;(g/L)$$

(4) $$PHA\;accumulation\;\left(\%\right)=\frac{Extracted\;quantity\;of\;PHA\;\left(g/L\right)}{Dry\;cell\;weight\;\left(g/L\right)}\times 100$$

(5) $$PHA\;productivity\;(Qp)=\frac{PHA\;final\;quantity\;(g/L)}{Fermented\;period\;(48\;h)}$$

### 2.5. PHA Extraction and Purification

The bacterial cells were collected after 48 h of fermentation. PHA was extracted by mechanically mixing the lyophilized bacterial biomass in chloroform, followed by treatment with sodium hypochlorite. The polymer was subsequently precipitated using methanol (80%) and recovered by filtration [41]. PHA polymer was further precipitated with 80% methanol, and the product was collected through vacuum filtration [42]. The resulting white PHA polymer was washed with the methanol–chloroform mixture and finally dried in a hot air oven at 60 °C for 48 h. The purified PHA was further selected for subsequent analytical characterization.

### 2.6. Analytical Methods

The chemical compositions of spent coffee ground waste biomass, including cellulose, hemicellulose, and lignin, were estimated using the Goering and Van Soest method [37]. Fermentable sugars (cellobiose, galactose, mannose, arabinose, and glucose) and other soluble metabolites (5-hydroxymethylfurfural and furfural) present in SCGO were quantified using an HPLC system (Agilent 1200, Palo Alto, CA, USA) equipped with a Shodex SH1011 column (Tokyo, Japan) and RI detector. Analysis was carried out using 10 mM H_2_SO_4_ as mobile phase at a flow rate of 0.5 mL/min and column temperature of 55 °C. Using an elemental nitrogen analyzer (Thermo Finnigan EA 1112, Waltham, MA, USA), the nitrogen content in the biomass was measured. With the aid of a nitrogen-to-protein conversion factor of 6.25 g protein/g of nitrogen, the protein concentration was determined. To determine the total phenolic content (TPC) of the extract, the standard Folin–Ciocalteu method was used [43]. Total flavonoid content (TFC) was determined by the aluminum chloride colorimetric method using rutin as the standard [44]. Briefly, SCGO extract (10 mg/mL) was added to aluminum chloride (AlCl_3_) solution and glacial acetic acid and was allowed to stand at room temperature for 40 min, after which it was diluted with ethanol, and absorbance was measured at 415 nm. The TFC was expressed in terms of mg rutin equivalents (RE) per g of dry extract. The reducing sugar (RS) content was measured using the dinitrosalicylic acid (DNS) method [45].

### 2.7. PHA Characterization

Fourier-transform infrared spectrometry (FTIR) (Cary 630 FTIR, Agilent, Santa Clara, CA, USA) in the spectral range of 4000–400 cm^−1^ and at 4 cm^−1^ resolution was used to analyze the functional group composition of produced PHA. X-ray diffraction (XRD) was used under standard operating conditions with an X-ray diffractometer system (D2 Phaser, Bruker, Berlin, Germany) to determine the crystallinity of produced PHA. The measurements were performed at room temperature, and the scans were taken between 10 and 50° at a 2θ range of a scanning rate of 1°/min.

Thermal properties of PHA were studied by Thermogravimetric Analysis (TGA; Hi-Res 2950, TA Instruments, New Castle, DE, USA) and Differential Scanning Calorimetry (DSC; DSC2920, TA Instruments, New Castle, DE, USA). The thermal stability of PHA was evaluated using TGA, where approximately 10 mg of the sample was heated from 20 to 500 °C at a rate of 20 °C/min in a nitrogen atmosphere. DSC was used to determine the glass transition (Tg) and melting temperature (Tm) of approximately 2 mg of the analyzed PHA at a heating rate of 10 °C/min and a cooling rate of 5 °C/min over a temperature region of −50–250 °C. All analyses were performed as described previously [46].

### 2.8. Statistical Analysis

The statistical analysis was done using GraphPad InStat software (version 3.06; GraphPad Software Inc., San Diego, CA, USA). One-way analysis of variance (ANOVA) was used to analyze experimental data to determine whether any significant differences existed between treatment groups. If a significant effect was detected, Tukey’s honestly significant difference (HSD) post hoc test was used for multiple pairwise comparisons among the group means. Each experiment was repeated three times, and results are shown as means ± SD (standard deviation). Differences were considered statistically significant at *p* < 0.05.

## 3. Results and Discussion

Due to increased production and consumption of coffee, coffee-waste generation has increased substantially. SCG production in South Korea increased about 1.6-fold, from 93,397 tonnes in 2012 to 149,038 tonnes in 2019 [46]. Despite this trend, limited attention has been given to the valorization of defatted coffee grounds to produce biopolymers. In this research, SCGO was subjected to different chemical pretreatments, and then enzymatic hydrolysis was conducted to evaluate their suitability for PHA production.

### 3.1. Compositional Analysis of Spent Coffee Ground (SCG) Biomass

The spent coffee grounds (SCG) biomass was first exposed to oil extraction in *n*-hexane under controlled conditions. The percentage extractable oil of SCG was 12.4%, with the fat percentage being 12.8% on a dry weight basis. Such values are within the range of 10–15% reported in prior research on SCG with identical composition [47]. The choice of solvent plays a major role in oil recovery, wherein non-polar solvents exhibit better performance than polar solvents. Extraction of SCG with hexane for 30 min has produced yields of 15.28% and 14.7%, which were higher than ethanol (13.1%) and methanol (7.5%) methods [48,49]. After oil extraction, SCGO contained potentially antimicrobial compounds, mainly polyphenols. To reduce the chances of their interference with enzyme hydrolysis and subsequent microbial fermentation of PHA, the SCGO was pre-extracted using 80% methanol before being subjected to chemical pretreatment.

Table 1 displays the chemical compositions of SCG waste biomass. Compositional analysis demonstrated that SCG contains hemicellulose, mainly mannans and galactans, at 39.3 g/100 g, which is substantially greater than the cellulose amount (9.3 g/100 g). SCG also has about 9% protein. SCG contains 22.5 g/100 g of lignin, which contributes to its high caloric content [50]. The chemical constituents of SCG used in this study were consistent with prior reports [15,51]. The differences in SCG are possibly explained by the variations in coffee bean origin and roasting conditions [52]. In general, SCG consists of polysaccharides (hemicellulose and cellulose) that approximately equal 48.6 g/100 g of dry SCG, emphasizing its potential as a promising feedstock for biochemicals, biofuels, and polyhydroxyalkanoates (PHAs).

### 3.2. Chemical Pretreatments of Defatted SCGO

Defatted SCGO consists of cellulose, which is bound in a hemicellulose–lignin structure, which limits access by enzymes and hinders its saccharification. In particular, process performance can be significantly enhanced by reducing the lignin content through an adequate pretreatment step before utilizing the SCGO as a feedstock to produce PHA. Thus, it is crucial to develop an effective pretreatment solution that breaks down the lignocellulosic framework and leaves sufficient lignification so that the holocellulose is more readily available and can be converted to PHA. The appropriateness of chemical pretreatments with acid (H_2_SO_4_), alkali (NaOH), and peracetic acid (PAA) was assessed to enhance the enzymatic digestibility of SCGO in the present research. Pretreatment of SCGO using sulfuric acid enhances the hydrolysis of the lignocellulosic matrix, leading to significant delignification and higher depolymerization of biomass. Nevertheless, during the process, inhibitory byproducts are formed, including furfural, HMF, and organic acids such as acetic and formic acids [15]. These compounds, especially furfural and HMF, can impede microbial growth and metabolism, thus hindering PHA generation [50].

**Figure 2:**
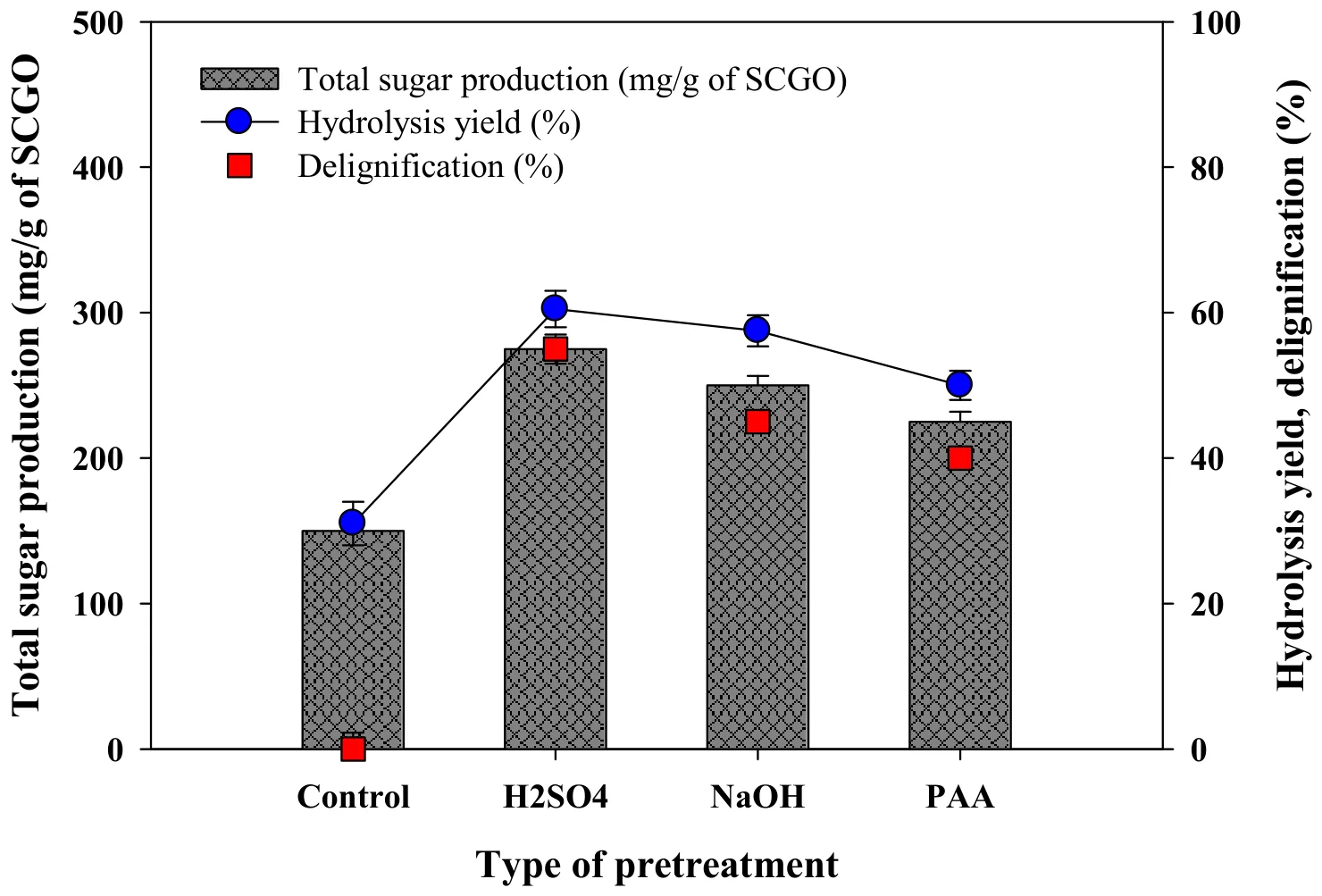
Effect of acid, alkaline, and PAA pretreatment on lignin removal, hydrolysis yield, and total reducing sugars production after enzymatic hydrolysis of SCGO (crude enzyme dosage 20 FPU/g of SCGO).

Alkaline pretreatment is particularly effective in reducing biomass crystallinity and promoting fiber swelling. Saponification of acetyl and uronic ester bonds improves enzyme access to the internal structure of the biomass and results in higher reducing sugar yields [51]. In addition, peracetic acid (PAA) is a potent oxidizing agent that interferes with lignin structure, cleaving side chains and β-aryl ester bonds and oxidizing aromatic units of lignin. The lignin fragments remaining after the depolymerization process are soluble in water, hence enhancing the availability of holocellulose to undergo enzymatic hydrolysis [32,33]. Nevertheless, PAA is a powerful oxidizer, and handling at industry scale will need appropriate safety measures and process control. The chemical pretreatments tested showed that acid and alkaline pretreatments performed better than PAA pretreatment in terms of delignification and hence made biomass more accessible to enzymatic hydrolysis. This effect caused acid pretreatment to yield the greatest reducing sugar product (around 275 mg/g biomass) and hydrolysis efficiency (around 60.5%). Comparatively, the alkaline and PAA pretreatments produced reducing sugars of approximately 250 and 225 mg/g of SCGO, with hydrolysis efficiencies of 57.5 and 50%, respectively. The results are presented in Figure 2.

The biochemical composition of SCGO was evaluated before and after alkaline pretreatment. Crude biomass composition before pretreatment was 9.3% cellulose, 39.3% hemicellulose, and 22.5% lignin. The alkaline-treated SCGO cellulose content increased to 38.5% while those of hemicellulose and lignin decreased to 16.83% and 9.6%, respectively. Cellulose accounted for about 54% of the quantified lignocellulosic content of the pretreated SCGO biomass based on the combined cellulose, hemicellulose, and lignin fractions. After pretreatment, approximately 52.5% of the SCGO remains as residual biomass. Hemicellulosic carbohydrates and other polysaccharides were the main part of the remaining fraction. These results show that alkaline pretreatment successfully enriched the cellulose fraction while removing the significant amount of hemicellulose and lignin. Cellulose recovery and lignin removal were 98% and 46%, respectively, for SCW treated with 4% KMnO_4_ and ultrasound for 20 min. The residual biomass yield after alkaline pretreatment was around 52.5% [53].

**Figure 3:**
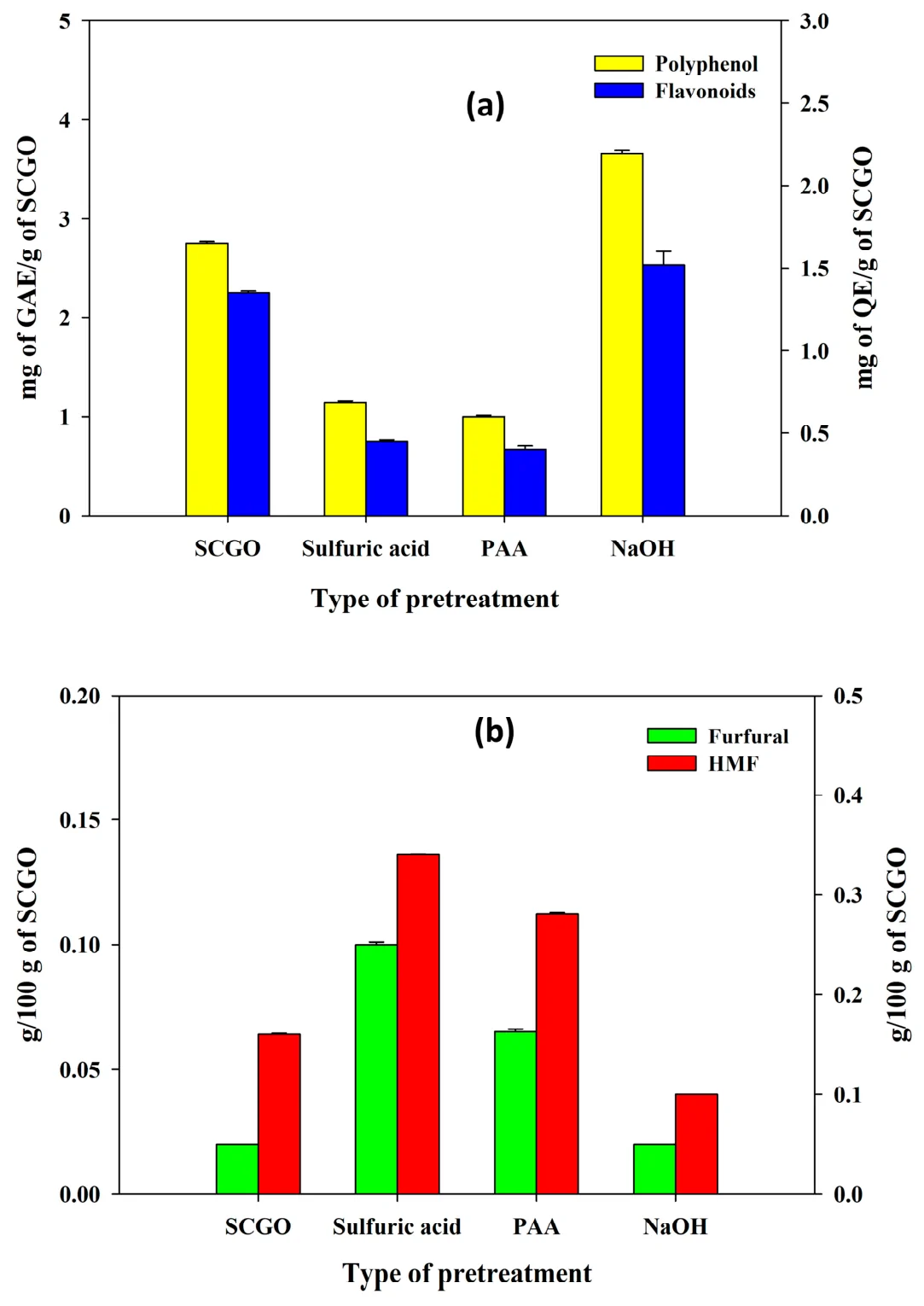
(**a**) Polyphenol and flavonoid contents and (**b**) furfural and hydroxymethylfurfural (HMF) contents before and after different chemical treatments of SCGO.

Lignocellulosic hydrolysates contain several microbial inhibitors originating from native biomass constituents and pretreatment-induced degradation reactions. Pretreatment can be useful for breaking up biomass and improving sugar recovery but can also cause the formation of inhibitory byproducts. These include weak organic acids, phenolic compounds, furfural, and HMF, which are known to negatively affect microbial growth, enzymatic hydrolysis, and fermentation performance [54,55]. Their composition and concentration depend on the feedstock characteristics and pretreatment severity, and they can substantially reduce microbial growth, substrate utilization, and product formation [55]. Furfural and HMF interfere with cellular redox balance, enzyme activity, energy metabolism, and DNA synthesis, resulting in prolonged lag phases and reduced fermentation performance in microorganisms such as *Saccharomyces cerevisiae* and *Cupriavidus necator* [11,13]. Similarly, lignin-derived phenolic compounds can disrupt cell membranes, induce oxidative stress, and inhibit key metabolic enzymes [56]. The concentrations of major inhibitors such as phenolic compounds, flavonoids, furfural, and HMF were recorded for different pretreatment procedures (Figure 3). During acid pretreatment, both furfural and HMF amounts increased relative to untreated SCGO, while there was a reduction in polyphenolic content. Alkaline pretreatment predominantly removes lignin and disrupts ester linkages, resulting in the release of phenolic compounds as the major inhibitory products [55]. This resulted in an increase in both polyphenol and flavonoid content of SCGO after alkaline pretreatment compared to the control in the three-stage detoxification process. Thus, there is a need to check the suitability of the obtained SCGO hydrolysates as a carbon source for microbial fermentation and subsequent PHA production by *C. necator*.

### 3.3. Enzymatic Hydrolysis of SCGO

In this study, the crude enzyme cocktail was produced using isolated *Streptomyces* sp. MDS comprised a set of lignocellulose-degrading enzymes comprising cellulolytic and hemicellulolytic activities [43]. The highest priority in the use of microbial enzyme preparation was to test the possibilities of using a cheap, in-house microbial enzyme system for saccharification of SCGO. Optimization of hydrolysis conditions and cellulase loading is necessary due to the substrate composition, hydrolysis kinetics, and process economics [57].

Optimizing enzymatic hydrolysis conditions, such as pH, temperature, substrate loading, and enzyme dosage is needed to improve saccharification efficiency and enable cost-effective, industrially viable biopolymer manufacture using SCGO. The experiments showed significant enzymatic hydrolysis of NaOH-pretreated SCGO at 50 °C and an initial buffer pH of 5.0.

**Figure 4:**
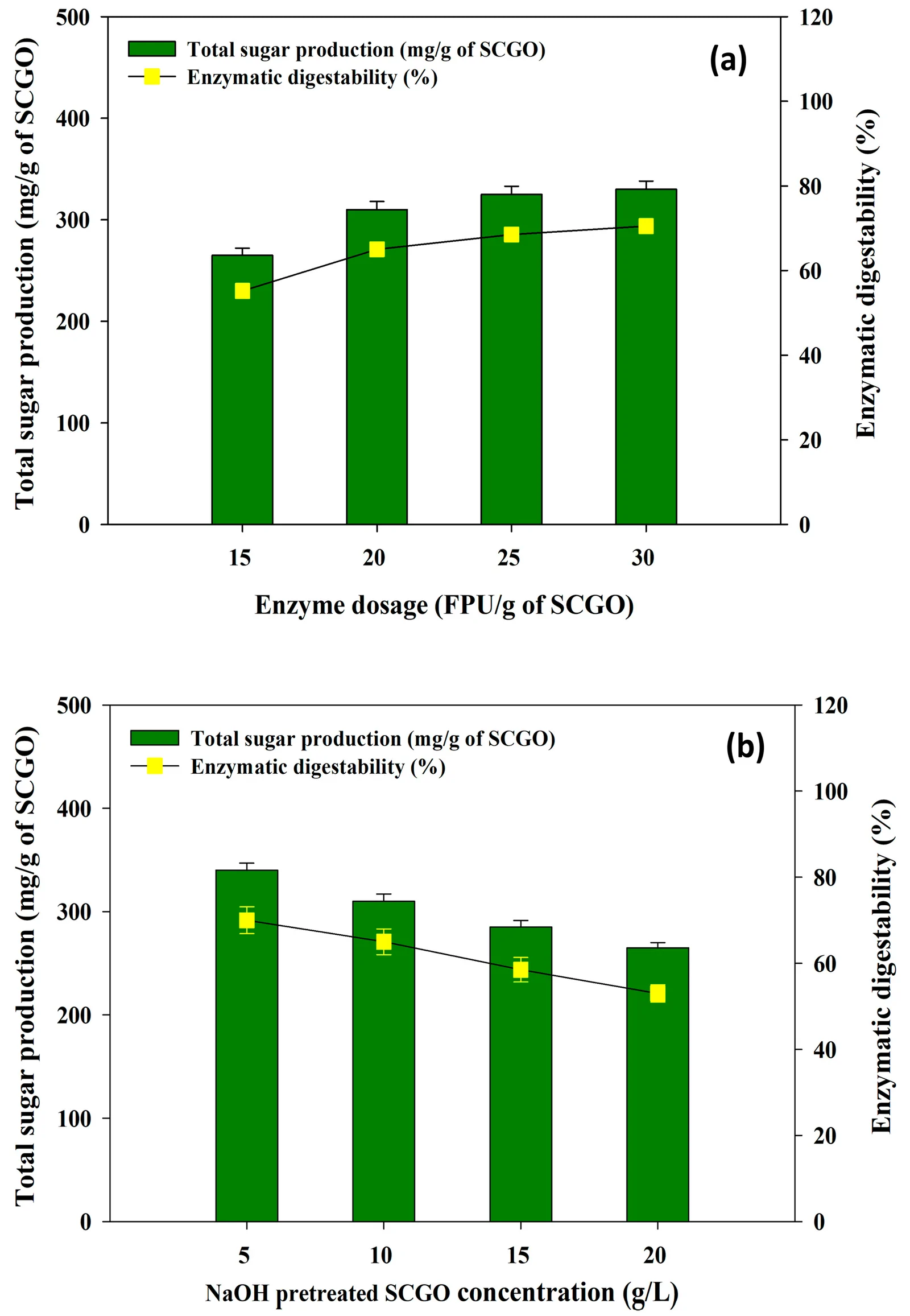
Effects of (**a**) enzyme loading (15, 20, 25, and 30 FPU/g SCGO) at a constant alkaline-pretreated SCGO concentration of 10 g/L and (**b**) alkaline-pretreated SCGO concentration (5, 10, 15, and 20 g/L) at a constant enzyme loading of 20 FPU/g SCGO on the enzymatic digestibility and total reducing sugar production from SCGO.

Effects of enzyme loading (15, 20, 25, and 30 FPU/g SCGO) at a constant alkaline-pretreated SCGO concentration of 10 g/L and alkaline-pretreated SCGO concentration (5, 10, 15, and 20 g/L) at a constant enzyme loading of 20 FPU/g SCGO on the enzymatic digestibility and total reducing sugar production from SCGO were assessed, respectively. The required cellulase dosage varies depending on the substrate type, hydrolysis time, and enzyme cost [38]. The highest level of saccharification was obtained at a substrate concentration of 10 g/L and an enzyme loading of 20 FPU/g of dry SCGO, which produced a TRS yield of 310 mg/g of SCGO (Figure 4a,b). However, further increase in the enzyme dose to 25 FPU/g and 30 FPU/g showed slight numerical increases in the TRS yield to 325 and 330 mg/g of SCGO, respectively. This phenomenon could be explained by the accumulation of end products such as glucose and cellobiose and their inhibitory effects on cellulase. Therefore, 20 FPU/g was selected as the optimum enzyme loading considering enzyme consumption and process economics. Increasing the solid loading of SCGO enhanced the concentration of reducing sugar up to 10% (*w*/*v*). Further increase in solid loading to 15% and 20% (*w*/*v*) resulted in total reducing sugar (TRS) yields of 285 and 265 mg/g SCGO, respectively. These results indicate that increasing the solid loading beyond 10% (*w*/*v*) did not notably improve sugar release (Figure 4b). Therefore, 10% (*w*/*v*) was selected as the ideal solid loading for subsequent experiments. The obtained TRS yield was similar to that previously obtained on the CaO_2_-pretreated lignocellulosic biomass, which produced about 325 mg/g of reducing sugar [58].

### 3.4. PHA Production Using Chemically Pretreated SCGO Enzymatic Hydrolysates

The use of SCGO to produce polyhydroxyalkanoate (PHA) is a potential solution to the problem of waste disposal and a sustainable path to the production of valuable biodegradable bioplastics [59,60,61]. Nevertheless, fermentable sugars are not the only components of SCGO, as it also contains high-molecular-weight melanoidins, alkaloids, and phenolic compounds, which can also exert antimicrobial effects [15].

**Figure 5:**
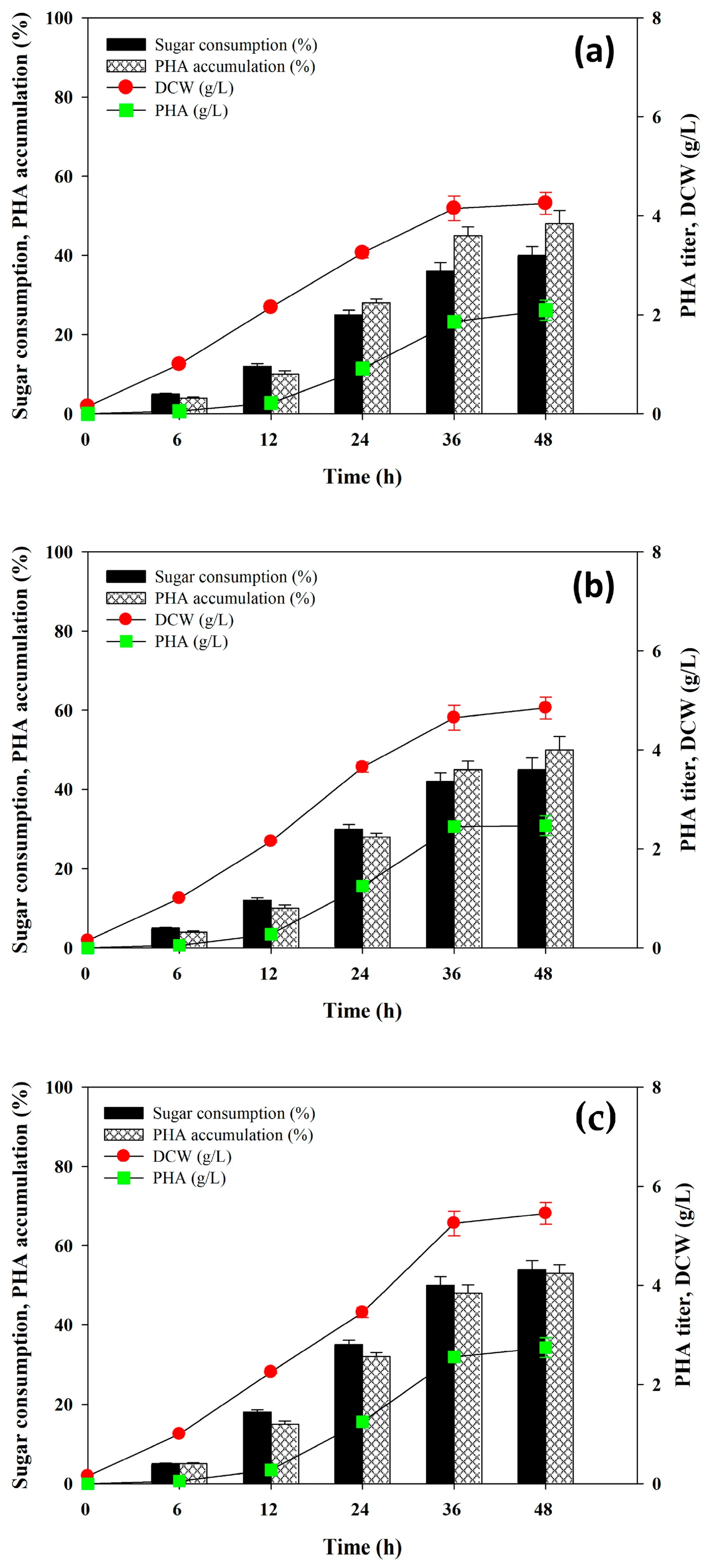
Sugar consumption, PHB production and growth parameters of *C. necator* using (**a**) acid-, (**b**) PAA- and (**c**) alkaline-pretreated SCGO enzymatic hydrolysates (each 20 g/L concentration) at different incubation times.

Moreover, chemical pretreatment produced fermentation inhibitors that negatively impact microbial growth and PHA production. Hence, to achieve efficient microbial transformations of SCGO into PHA, it is important to evaluate the appropriateness of various chemical pretreatment strategies. *C. necator* was used in batch fermentation in the presence of enzymatic hydrolysates of acid-, alkali-, and peracetic acid (PAA)-pretreated SCGO (20 g/L) as the sole carbon source in the fermentation. Figure 5 shows biomass formation, PHA accumulation, and PHA titers obtained using each hydrolysate. No detoxification was applied to any of the hydrolysates. Among the methods tested, acid pretreatment yielded the best reducing sugar production following enzymatic hydrolysis. However, hydrolysates of H_2_SO_4_-pretreated and PAA-pretreated SCGO had low sugar utilization (40% and 45%), lower cell growth (4.25 g/L and 4.85 g/L), and PHA production (2.09 g/L and 2.47 g/L), respectively (Figure 5a,b). By comparison, greater consumption of sugars (58%), better biomass growth (DCW of 5.65 g/L), and a more pronounced PHB titer (3.06 g/L) were achieved by alkali-pretreated SCGO hydrolysate (Figure 5c). This was probably because of the presence of inhibitory compounds, predominantly phenolic and furanic compounds, which inhibited microbial metabolism and PHA biosynthesis [62]. The overall PHA yield from raw SCGO and PHA yield on consumed sugars for alkali (20.1 mg PHA/g raw SCGO and 0.263 g/g of consumed sugars), whereas, for acid and PAA, overall PHA yield (14.2 and 13.1 mg PHA/g raw SCGO) and PHA yield on consumed sugars (0.278 and 0.241 g/g of consumed sugars) were measured, respectively [63]. Alkali-pretreated SCGO enzymatic hydrolysates were determined to better support *C. necator* growth and PHA production, so all experimental work was carried out to maximize PHA synthesis by optimizing pretreatment conditions.

Pretreatment is usually the most expensive step of biorefinery processes, making up to 40% of the total production cost [64]. Alkaline pretreatment enables biomass delignification by breaking acetyl and uronic ester crosslinking of lignin and hemicellulose, swelling crystalline cellulose, and increasing the external surface area [65]. In the current work, although alkaline pretreatment produced a lower total sugar percentage than acid and PAA pretreatment in the initial session, better results in enhancing *C. necator* growth and PHA production were observed. The process variables, including the influence of the alkali concentration, solid loading, temperature, and residence time, were studied to achieve the maximum delignification (55%) and saccharification yield up to 305 mg/g of SCGO, observed at 2% NaOH, 10% solid loading at 100 °C and 4 h of incubation. One of the most important parameters affecting the pretreatment efficiency was found to be residence time. Extending the treatment period from 3 h to 4 h considerably enhanced biomass hydrolysis as well as saccharification of SCGO. The chosen pretreatment conditions are used in further experiments.

### 3.5. PHA Production Using Alkaline Pretreated SCGO Hydrolysates by C. necator

Spent coffee ground is a potential renewable and sustainable source of PHA production, which aligns with the requirements of the United Nations Sustainable Development Goals, specifically Goal 12 [66]. The concentration of substrate is an important operational parameter, and it directly regulates microbial metabolism, substrate uptake, microbial growth, and PHA biosynthesis. *Cupriavidus necator* was used in batch fermentations with alkaline-pretreated SCGO enzyme hydrolysates as the sole carbon source at the following concentrations of 10, 20, 30, and 40 g/L. Table 2 demonstrates the biomass formation, intracellular PHA accumulation, and PHA yield at different concentrations of the SCGO hydrolysates. The SCGO hydrolysates could be effectively assimilated up to 20 g/L, with subsequent improved cellular growth and PHA accumulation. The details of sugar assimilation, *C. necator* growth, and PHA production parameters at different SCGO concentrations have been summarized (Table 2). Nonetheless, further addition of substrate concentration resulted in reduced sugar assimilation, reduced biomass growth, and further improvement in PHA output. The lower yield at increased hydrolysate concentrations can probably be explained by the presence of inhibitory compounds such as polyphenols and other bioactive molecules, which may negatively impact microbial metabolism and PHA biosynthesis [67]. Alkaline pretreatment predominantly removes lignin and disrupts ester linkages, resulting in the release of phenolic compounds as the major inhibitory products [55]. To further enhance PHA production by *C. necator* using SCGO alkaline hydrolysates, further studies were undertaken.

The use of defined synthetic sugar mixtures for comparative fermentations, along with intentional addition of known inhibitors, would be beneficial for quantitative differentiation of both sugar concentrations and inhibitor toxicity on PHA production in future studies.

### 3.6. Enhanced PHA Accumulation Under Stress Conditions

The production of PHA by microbes tends to trigger intracellular PHA accumulation as a microbial survival mechanism in stressful environments. Besides nutrient limitation, the use of external stressors has also been reported extensively in stimulating PHA biosynthesis. This stress leads to metabolic reprogramming or altered activity of major enzymes associated with bacterial growth and PHA production [68]. The effect of external stress on the study was examined by adding ethanol and methanol to the fermentation medium. Besides that, varying levels of NaCl (5, 10, and 15 g/L) were tested in the presence of SCGO hydrolysates in the production medium.

**Figure 6:**
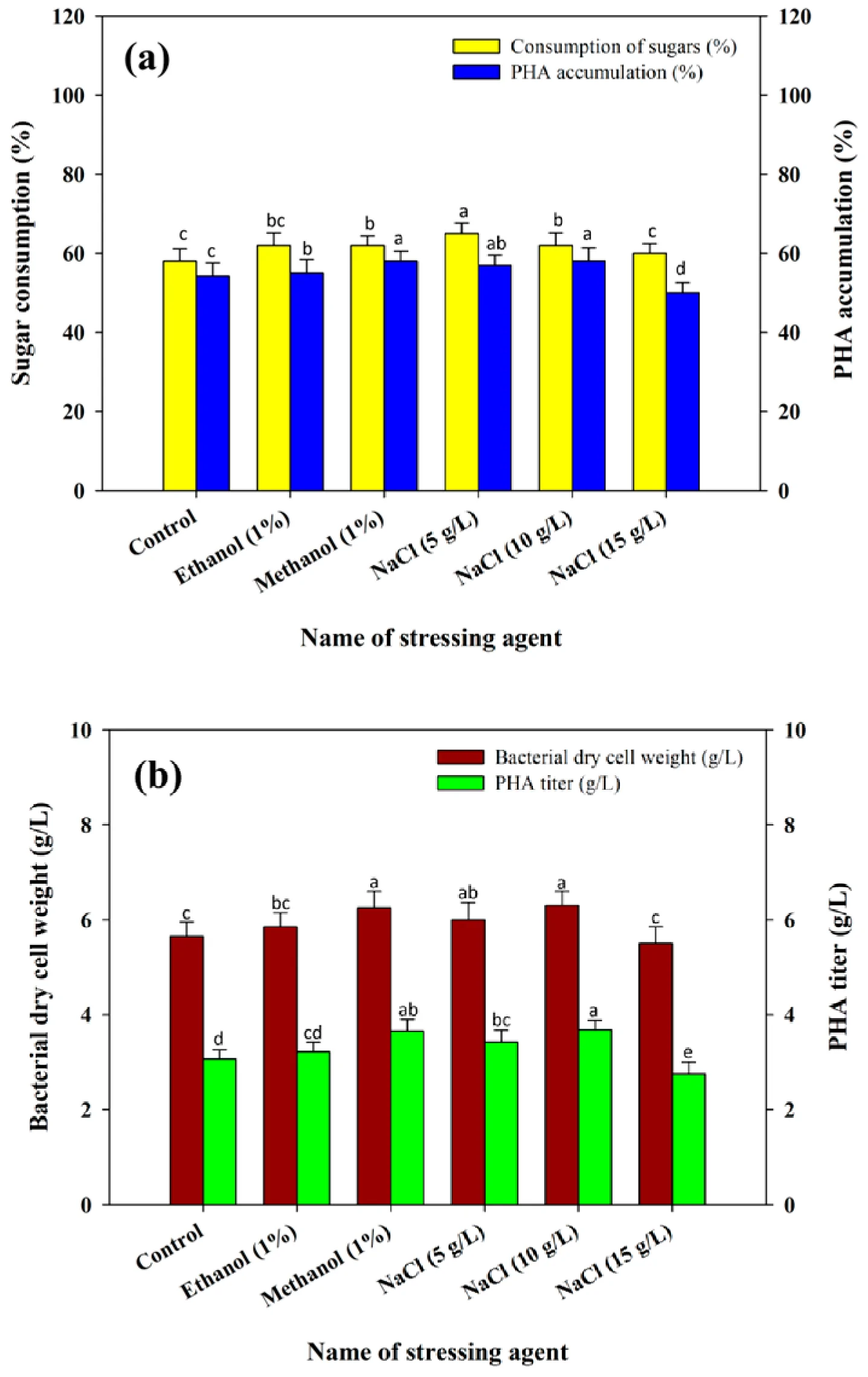
Effect of applying stress conditions with alkaline-pretreated SCGO hydrolysates (20 g/L) on (**a**) sugar consumption and PHA accumulation; (**b**) *Cupriavidus necator* growth and PHA production. The results (bars) are presented as mean ± SD (*n* = 3). Significant differences between treatments at *p* < 0.05 are defined using Tukey’s HSD test, with different lowercase letters.

The addition of 1% (*v*/*v*) methanol and 10 g/L NaCl substantially improved bacterial growth, leading to a 12% increase in biomass and a 20% enhancement in PHA production relative to the control (Figure 6). These findings are in line with Passanha et al. [69], who found a 30% increase in PHA production by *C. necator* when 9 g/L NaCl was added to the production medium. Taken together, the current findings reveal that external stress with a 1% methanol supplement and moderate osmotic stress in fermentation media with SCGO hydrolysates is advantageous in promoting PHA production in *C. necator*. Among the individual factors evaluated using a one-factor-at-a-time approach, supplementation with 10 g/L NaCl resulted in the highest PHA accumulation.

### 3.7. Enhanced PHA Accumulation by Supplying Complex Nutrient Supplements

**Figure 7:**
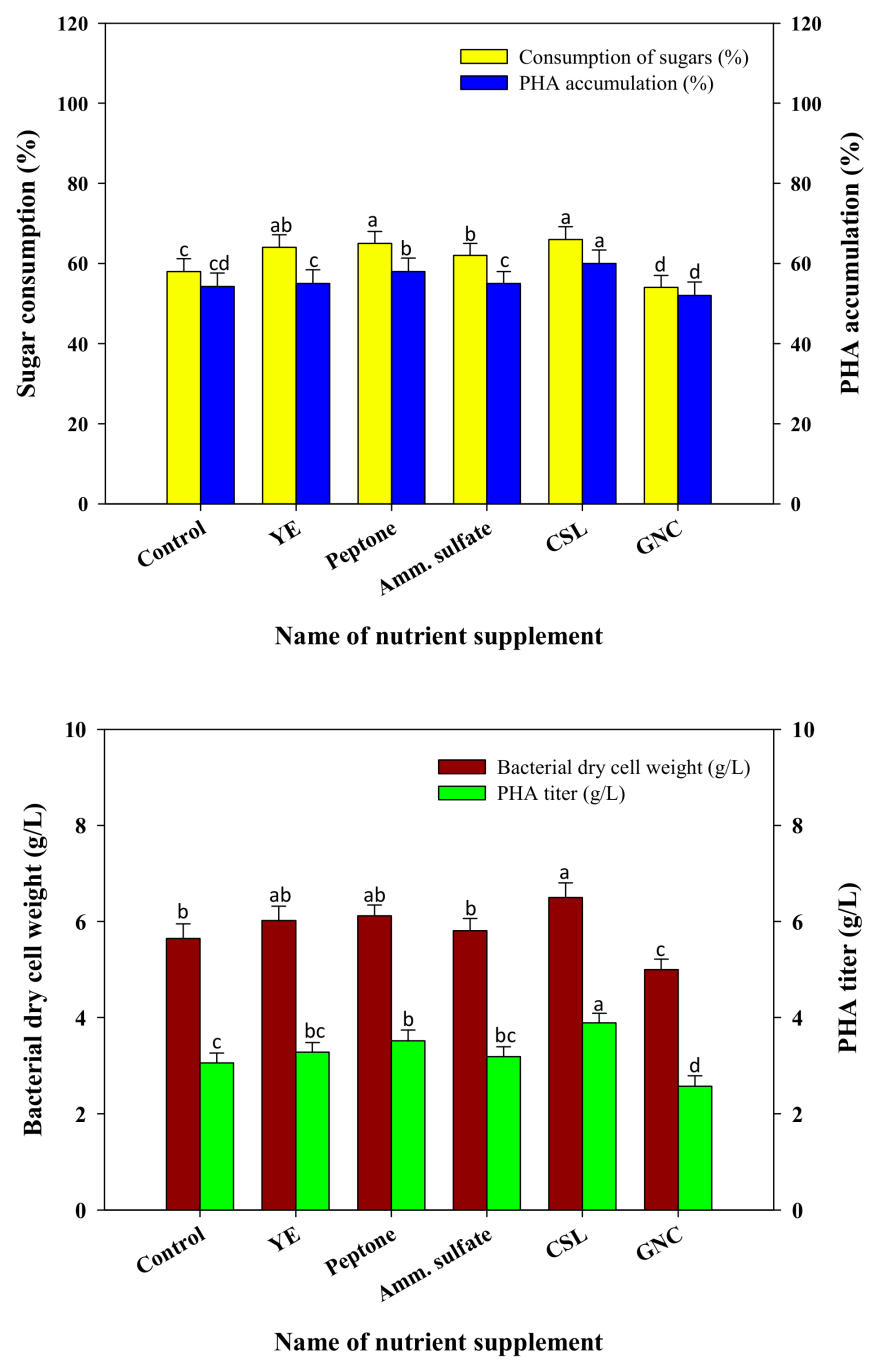
Effects of supplying various complex nutrient supplements with alkaline pretreated SCGO hydrolysates (20 g/L concentration) on sugar consumption, *Cupriavidus necator* growth and PHA production. The results (bars) are presented as mean ± SD (*n* = 3). Significant differences between treatments at *p* < 0.05 are defined using Tukey’s HSD test, with different lowercase letters.

Production of PHA by bacteria leads to the build-up of PHA during abundant carbon and inadequate levels of nitrogen and phosphorus, elevating stress resistance. Nitrogen controls cell growth in the initial phase and protein synthesis, and then PHA accumulates when the nutrient is limited, whereas phosphorus facilitates necessary cellular and metabolic functions [14,70]. Hence, it is essential to regulate nitrogen and phosphorus as closely as possible to maximize PHA production. In this study, yeast extract, peptone, ammonium sulfate, and inexpensive supplements, corn steep liquor (CSL) and groundnut cake, were tested as a source of nitrogen and phosphorus in the fermentation media containing alkaline-pretreated SCGO enzymatic hydrolysates. Peptone and yeast extract improved sugar uptake and bacterial growth better than ammonium sulfate. With peptone, meaningful increases in biomass (11%), PHA accumulation (8%), and PHA yield (15%) over the control were observed. The CSL supplementation caused substantial sugar consumption (66%), biomass production (DCW; 6.5 g/L), and PHA production (3.89 g/L). The results are shown in Figure 7. Therefore, the process can be made eco-friendly, cost-efficient, and viable by adding the CSL to the fermentation media. These results are consistent with earlier reports that showed a substantial increase in cell growth and PHB generation in recombinant *E. coli* receiving yeast extract, which is attributed to enhanced intracellular NADPH generation by biosynthetic aid of reduced amino acid demand [71,72]. This implies that, under these conditions, nutrient availability is more important than stress induction.

Stress conditions primarily serve as physiological modulators, and CSL provides assimilable nitrogen, amino acids, vitamins, minerals, growth factors and metabolic activity in cells. Thus, while it is possible that stress-induced phenomena caused PHA accumulation, the significant performance improvement by the nutrient-supplemented treatment was most noteworthy. The obtained results of biomass production and PHA synthesis by *C. necator* using SCGO hydrolysates with CSL are comparable with the results obtained with *Burkholderia cepacia* using SCG hydrolysate, where biomass yield of 5.5 g/L; PHA accumulation (56%) and PHA titer of about 3.1 g/L were recorded [65]. Other studies reported using SCG-derived oil for PHA production, mainly by *Cupriavidus necator* and *Pseudomonas resinovorans* [70,71]. The current status of PHA production using SCGO hydrolysates is in the initial stage; thus, more fundamental research is still required. This study identified the best-performing individual treatment under OFAT conditions. Further optimization using multifactorial experimental designs is warranted to determine the optimal combination of stress and nutrient factors to ensure maximum microbial performance and PHA production.

### 3.8. Characterization of Produced PHA

**Figure 8:**
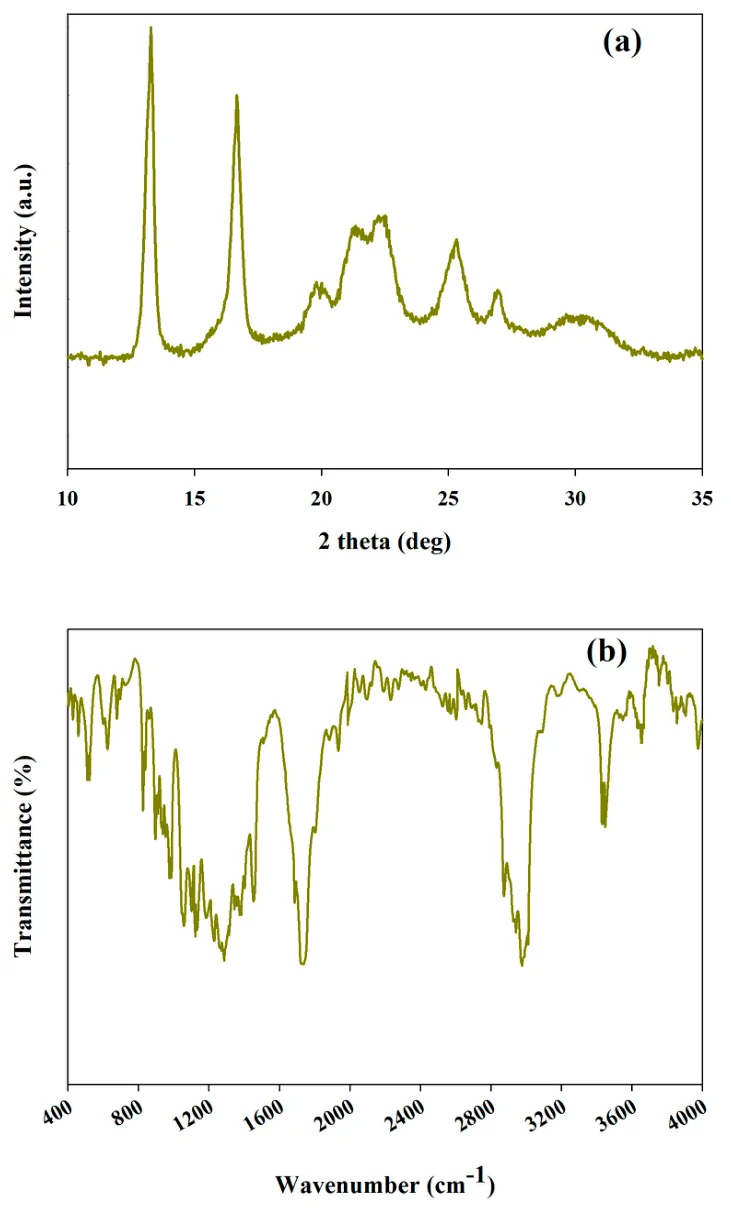
(**a**) XRD pattern and (**b**) FTIR spectrum of the produced PHA by *C. necator* using alkaline-pretreated SCGO enzymatic hydrolysates.

The XRD pattern reveals clear diffraction peaks at 2θ = 13.42° and 16.18°, indicating that the material produced is crystalline PHB. The observed reflections at 2θ = 21.24° and 22.88° are typical reflections of crystalline planes that belong to the α-PHB phase, namely, orthorhombic. Moreover, the peaks of 2θ = 25.68° and 27.65° denote the existence of a partially crystalline structure (Figure 8a). The XRD results are consistent with previous studies on PHB, such as PHB synthesized by *C. necator* using sucrose as a carbon source [73], PHB synthesized by *C. necator* using rice waste biomass [40], and PHB synthesized by *C. necator* using red grape pomace [74]. The FTIR spectrum of the obtained PHB is shown in Figure 8b, and the distinctive absorption bands are observed. The band at 3440 cm^−1^ is associated with -OH groups, whereas the one at a minimum of 2974 cm^−1^ relates to aliphatic C-H stretching [75,76]. PHB is confirmed by the strong absorption at 1720 cm^−1,^ suggesting the presence of the (C=O) ester carbonyl group. Other bands at 1280–1470 and 1045 cm^−1^ correspond to bands of -CH and C-O-C, respectively [74,77,78,79]. Additional peaks at 978–513 cm^−1^ also indicate the presence of ester linkages.

### 3.9. TGA and DSC Analysis

**Figure 9:**
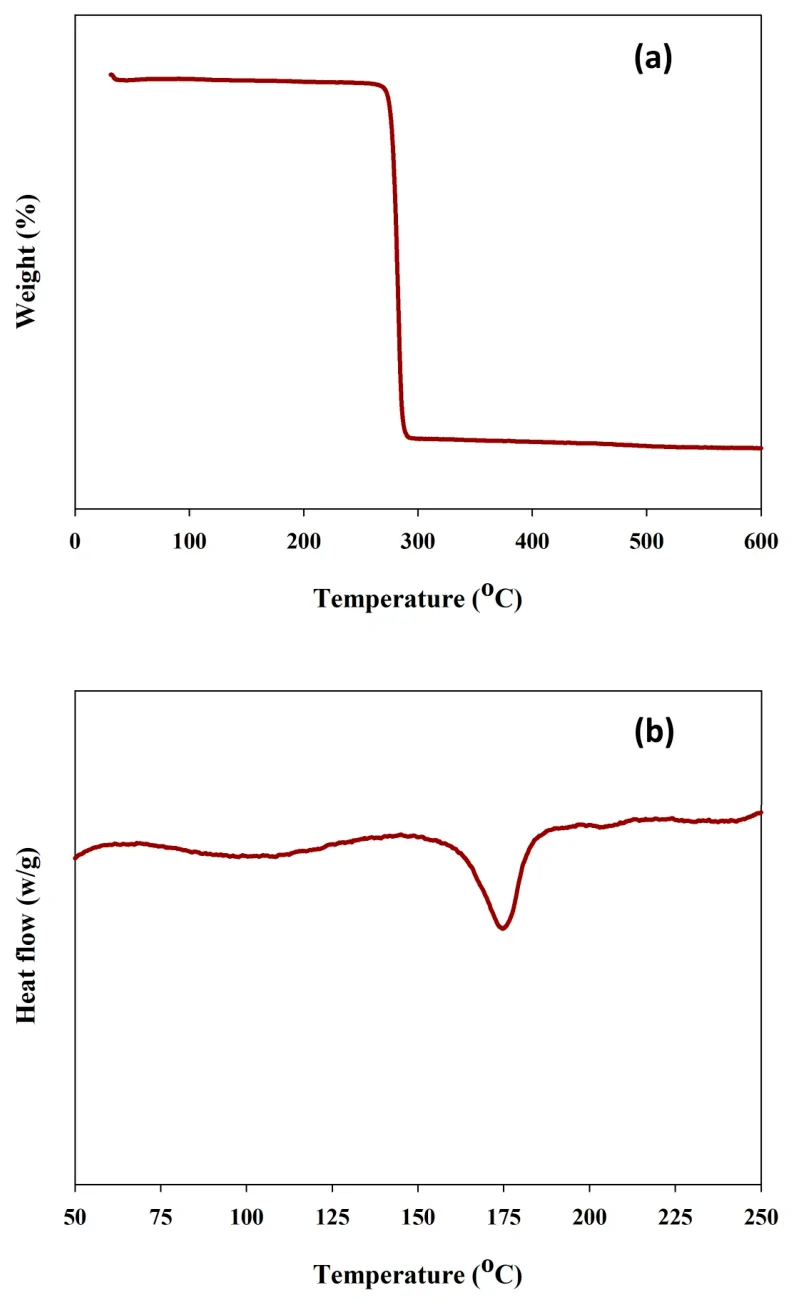
(**a**) Thermogravimetric analysis (TGA) and (**b**) differential scanning calorimetry (DSC) analysis of poly-(3-hydroxybutyrate) (PHB) produced by *C. necator* from alkaline-pretreated SCGO enzymatic hydrolysates.

Polyhydroxybutyrate (PHB), a well-known biodegradable and biocompatible microbial polyester, can be considered as a bio-based substitute for petroleum plastics. Standard TGA and DSC analytical techniques were employed to perform an exhaustive study of the thermal behavior of *C. necator*-produced PHA from SCGO hydrolysates for processing, industrial use, and thermal recycling [80]. TGA measures mass loss as a function of temperature and determines thermal stability and degradation temperature; DSC measures heat flow and is used to detect physical phase changes such as the glass transition temperature, crystallization temperature, and melting temperature [81,82]. When subjected to TGA, the thermogram exhibited a single-step degradation profile, and thermal degradation began at 290.6 °C; thus, it was associated with high thermal resistance (Figure 9a). The mass loss observed is due to cleavage of ester linkages via a β-elimination reaction. The Td value of the bacterially produced PHB was found to be higher than the value reported in other studies [82,83,84]. Tm of PHB in our study was about 174.2 °C, which is very close to the Tm (178 °C) of the standard PHB sample (Figure 9b) [40,83]. Based on the thermal study, it is inferred that the PHB produced by *C. necator* is of good quality and has higher thermal stability, making it more resistant to thermal degradation.

## 4. Technical Challenges and Future Research Directions

Despite the potential of spent coffee grounds (SCG) as a sustainable feedstock for PHA production, several key challenges must be addressed before industrial implementation:

(1) The highest priority is improving carbon utilization efficiency. Although alkaline pretreatment generated fermentable sugars, the predominant sugars in hemicellulose, mannose and galactose, which constitute a significant proportion of the SCGO hydrolysate, were not efficiently utilized by *Cupriavidus necator*. Therefore, metabolic engineering to improve the conversion efficiency of these sugars to PHA represents the most important research direction for increasing carbon conversion efficiency, PHA yield, and overall process productivity. Future research should focus on metabolic engineering of *Cupriavidus necator* or the development of robust microbial consortia capable of efficiently assimilating mixed sugars, which can increase carbon conversion efficiency and PHA production.
(2) When efficient sugar utilization is attained, scale-up of the process becomes the next major challenge. Optimizing and scaling up oxygen transfer, mixing hydrolysates, handling variable feedstocks, advanced detoxification, enzyme use, sterilization, and polymer recovery are essential issues that need to be addressed for industrial implementation.
(3) To understand the costs associated with PHA production, a complete techno-economic/life-cycle assessment and process-scale mass balance analysis should be conducted to compare these costs and environmental impacts with conventional plastics. These were not within the scope of this study but were marked as priorities for future work.
(4) Integrating PHA production within SCG biorefinery offers an additional opportunity to improve process economics and resource efficiency. Sequential recovery of coffee oil, phenolic compounds, PHAs, and lignin-rich residues can maximize resource utilization, diversify revenue streams, and enhance the sustainability of coffee waste valorization within a circular bioeconomy framework, therefore improving the overall economic viability. These considerations favor a more balanced view of the possible industrial application of the proposed process and sustainable bioplastics production.

## 5. Conclusions

The research demonstrated the effective hydrolysis of residual SCG biomass following oil and phenolic-compounds extraction through chemical pretreatments and enzymatic saccharification. Among the evaluated approaches, optimized alkaline pretreatment provided suitable SCGO hydrolysates for PHA production by *Cupriavidus necator*. Under optimized fermentation conditions, *C. necator* using alkaline-pretreated SCGO hydrolysates supplemented with corn steep liquor resulted in the maximum accumulation of PHA of 60%, with a high cell mass of 6.5 g/L and PHA titer of 3.89 g/L in 48 h. These findings demonstrate the potential of SCG as low-cost feedstock for microbial PHA production. However, techno-economic analysis, life-cycle assessment, pilot-scale validation, and improved sugar utilization within an integrated SCG biorefinery paradigm are vital. To determine industry relevance, future work should focus on cost-cutting methods such as detoxification and fermentation optimization by designing suitable bioreactors to enhance PHA production. The incorporation of SCG valorization into integrated biorefinery systems will improve the economic viability and efficiency of the process in a circular bioeconomy. There is also a need to carry out detailed techno-economic studies to determine the production costs, scalability, and market potential of the resultant PHA for use as a food packaging material.

## Figures and Tables

**Table 1:** Chemical composition of spent coffee ground (SCG) used in this study (g/100 g of dry SCG).

Name of Component	Chemical Composition (%)
Dry solid matter	96.8 ± 3.25
Proteins	9.1 ± 0.84
Fats/Lipids	12.4 ± 0.92
Lignin	22.5 ± 1.10
Total sugars	48.6 ± 2.12
Cellobiose	2.85 ± 0.05
Glucose	6.45 ± 0.25
Mannose	16.8 ± 0.35
Galactose	22.5 ± 0.44
Arabinose	ND
Total polyphenols	2.75 ± 0.01
HMF	0.16 ± 0.001
Furfural	ND
Ashes	4.58 ± 0.12

Values represent the mean of three independent experiments; (±) standard error (SE). Differences were analyzed by one-way ANOVA with Tukey–Kramer multiple comparisons test. HMF—hydroxymethylfurfural; ND—not detected.

**Table 2:** Results of PHA production using alkaline-pretreated SCGO hydrolysates at different concentrations by *C. necator*.

Parameters Studied	SCGO Hydrolysates Concentration (g/L)			
	10	20	30	40
Sugar utilization of SCGO hydrolysates (%)	75	58	52	40
Bacterial dry cell weight (g/L)	3.75 ± 0.16	5.65 ± 0.25	7.06 ± 0.32	7.53 ± 0.34
PHA accumulation (%)	60.0 ± 2.45	54.2 ± 1.90	52.4 ± 1.65	50.0 ± 1.50
PHA titer (g/L)	2.25 ± 0.12	3.06 ± 0.14	3.70 ± 0.16	3.75 ± 0.15
Residual biomass (g/L)	1.50 ± 0.08	2.59 ± 0.11	3.36 ± 0.16	3.78 ± 0.18
PHA yield (g/g of sugar)	0.300 ± 0.001	0.255 ± 0.001	0.237 ± 0.001	0.234 ± 0.001
Q_p_ g PHA/L/h	0.046 ± 0.001	0.063 ± 0.001	0.077 ± 0.002	0.078 ± 0.003

Values are the mean of three experiments; (±) standard error (SE) by one-way ANOVA with Tukey–Kramer Multiple Comparisons Test.

## Data Availability

The raw data supporting the conclusions of this article will be made available by the authors on request.
